# Supplementary material for: Improvement in Quality of Life with Pelvic Floor Muscle Training and Biofeedback in Patients with Painful Bladder Syndrome/Interstitial Cystitis
Source: J Clin Med. 2021 Feb 19;10(4):862. doi: 10.3390/jcm10040862 (PMC7922867; doi:10.3390/jcm10040862)
Supplement: Supplementary file 1 [file jcm-10-00862-s001.pdf]

Supplementary Documentation: Health-Related Quality of Life Questionnaire Used: SF - 36.

HOSPITAL CLÍNICO UNIVERSITARIO DE SALAMANCA

FACULTAD DE MEDICINA

SERVICIO DE UROLOGÍA

Sacyl

Esta encuesta tiene por objetivo conocer y valorar el Estado de Salud y Calidad de Vida Relacionada con la Salud en pacientes ATENDIDOS POR CISTOPATÍA INTERSTICIAL O SÍNDROME DE VEJIGA DOLOROSA.<br>El cuestionario es anónimo. Los datos e información contenidos son confidenciales y secretos y su tratamiento/proceso será únicamente estadístico. Su uso se destina exclusivamente a los objetivos de esta investigación.<br>Las preguntas que siguen se refieren a lo que usted piensa sobre su salud. Sus respuestas permitirán saber cómo se encuentra usted y hasta qué punto es capaz de hacer sus actividades habituales. Conteste cada pregunta tal como se indica. Si no está seguro/a de cómo responder a una pregunta, por favor conteste lo que le parezca más cierto.<br>**Gracias por su colaboración.**

CUESTIONARIO

NºHC: _________

Sexo: ☐ Varón ☐ Mujer **Edad:** _______________años

PROFESIÓ/OCUPACIÓN: ____

………………………………………………………………………………………

ESTILO DE VIDA:

Consumo de tabaco: ………………………………paquetes/año.

Exposición a anilinas………………………………………………………………………..

Exposición a fenacetina, clornafazina……………………………………………….

Exposición a ciclofosfamida……………………………………………………………………

Exposición a radiación…………………………………………………………………

Otros

Nivel de estudios: ☐ **Ninguno**

☐ Estudios 1os

☐ Estudios medios (Bachillerato, Formación Profesional)

☐ Estudios superiores o universitarios

Comorbilidad: ¿padecen algún tipo de enfermedad o dolencia?

☐ No

☐ Sí. **Indicar:** ☐ Enfermedades del corazón

☐ Tensión arterial alta

☐ Asma o enfermedad pulmonar

☐ Diabetes

☐ Úlcera o enfermedad estomacal

☐ Enfermedad intestinal

☐ Enfermedad de los riñones

☐ Enfermedad del SNC

☐ Anemia u otra enfermedad de la sangre

□ Cáncer
□ Depresión
□ Enfermedad locomotora o articular
□ Otro problema médico: _
____

VRS MARQUE UNA SOLA RESPUESTA

1. En general, usted diría que su salud es:
1 □ Excelente
2 □ Muy buena
3 □ Buena
4 □ Regular
5 □ Mala

2. ¿Cómo diría que es su salud actual, comparada con la de hace un año?
1 □ Mucho mejor ahora que hace un año
2 □ Algo mejor ahora que hace un año
3 □ Más o menos igual que hace un año
4 □ Algo peor ahora que hace un año
5 □ Mucho peor ahora que hace un año

LAS SIGUIENTES PREGUNTAS SE REFIEREN A ACTIVIDADES O COSAS QUE USTED PODRÍA HACER EN UN DÍA NORMAL.

3. Su salud actual, ¿le limita para hacer esfuerzos intensos, tales como correr, levantar objetos pesados, o participar en deportes agotadores?
1 □ Sí, me limita mucho
2 □ Sí, me limita un poco
3 □ No, no me limita nada

4. Su salud actual, ¿le limita para hacer esfuerzos moderados, como mover una mesa, pasar la aspiradora, jugar a los bolos o caminar más de una hora?
1 □ Sí, me limita mucho
2 □ Sí, me limita un poco
3 □ No, no me limita nada

5. Su salud actual, ¿le limita para coger o llevar la bolsa de la compra?
1 □ Sí, me limita mucho
2 □ Sí, me limita un poco
3 □ No, no me limita nada

6. Su salud actual, ¿le limita para subir varios pisos por la escalera?
1 □ Sí, me limita mucho
2 □ Sí, me limita un poco
3 □ No, no me limita nada

7. Su salud actual, ¿le limita para subir un solo piso por la escalera?
1 □ Sí, me limita mucho
2 □ Sí, me limita un poco
3 □ No, no me limita nada

8. Su salud actual, ¿le limita para agacharse o arrodillarse?
1 □ Sí, me limita mucho
2 □ Sí, me limita un poco
3 □ No, no me limita nada

9. Su salud actual, ¿le limita para caminar un kilómetro o más?
1 □ Sí, me limita mucho
2 □ Sí, me limita un poco
3 □ No, no me limita nada

10. Su salud actual, ¿le limita para caminar varias manzanas (varios centenares de metros)?
1 □ Sí, me limita mucho
2 □ Sí, me limita un poco
3 □ No, no me limita nada

11. Su salud actual, ¿le limita para caminar una sola manzana (unos 100 metros)?
1 □ Sí, me limita mucho
2 □ Sí, me limita un poco
3 □ No, no me limita nada

12. Su salud actual, ¿le limita para bañarse o vestirse por sí mismo?
1 □ Sí, me limita mucho
2 □ Sí, me limita un poco
3 □ No, no me limita nada

LAS SIGUIENTES PREGUNTAS SE REFIEREN A PROBLEMAS EN SU TRABAJO O EN SUS ACTIVIDADES COTIDIANAS.

13. Durante las 4 últimas semanas, ¿tuvo que reducir el tiempo dedicado al trabajo o a sus actividades cotidianas, a causa de su salud física?
1 □ Sí
2 □ No

14. Durante las 4 últimas semanas, ¿hizo menos de lo que hubiera querido hacer, a causa de su salud física?
1 □ Sí
2 □ No

15. Durante las 4 últimas semanas, ¿tuvo que dejar de hacer algunas tareas en su trabajo o en sus actividades cotidianas, a causa de su salud física?
1 □ Sí
2 □ No

16. Durante las 4 últimas semanas, ¿tuvo dificultad para hacer su trabajo o sus actividades cotidianas (por ejemplo, le costó más de lo normal), a causa de su salud física?
1 □ Sí
2 □ No

17. Durante las 4 últimas semanas, ¿tuvo que reducir el tiempo dedicado al trabajo o a sus actividades cotidianas, a causa de algún problema emocional (como estar triste, deprimido o nervioso?

1 ☐ Sí
2 ☐ No

18. Durante las 4 últimas semanas, ¿hizo menos de lo que hubiera querido hacer, a causa de algún problema emocional (como estar triste, deprimido o nervioso)?
1 ☐ Sí
2 ☐ No

19. Durante las 4 últimas semanas, ¿no hizo su trabajo o sus actividades cotidianas tan cuidadosamente como de costumbre, a causa de algún problema emocional (como estar triste, deprimido o nervioso)?
1 ☐ Sí
2 ☐ No

20. Durante las 4 últimas semanas, ¿hasta qué punto su salud física o los problemas emocionales han dificultado sus actividades sociales habituales con la familia, los amigos, los vecinos u otras personas?
1 ☐ Nada
2 ☐ Un poco
3 ☐ Regular
4 ☐ Bastante
5 ☐ Mucho

21. ¿Tuvo dolor en alguna parte del cuerpo durante las 4 últimas semanas?
1 ☐ No, ninguno
2 ☐ Sí, muy poco
3 ☐ Sí, un poco
4 ☐ Sí, moderado
5 ☐ Sí, mucho
6 ☐ Sí, muchísimo

22. Durante las 4 últimas semanas, ¿hasta qué punto el dolor le ha dificultado su trabajo habitual (incluido el trabajo fuera de casa y las tareas domésticas)?
1 ☐ Nada
2 ☐ Un poco
3 ☐ Regular
4 ☐ Bastante
5 ☐ Mucho

LAS PREGUNTAS QUE SIGUEN SE REFIEREN A CÓMO SE HA SENTIDO Y CÓMO LE HAN IDO LAS COSAS DURANTE LAS **4 ÚLTIMAS SEMANAS**. EN CADA PREGUNTA RESPONDA LO QUE SE PAREZCA MÁS A CÓMO SE HA SENTIDO USTED.

23. Durante las 4 últimas semanas, ¿cuánto tiempo se sintió lleno de vitalidad?
1 ☐ Siempre
2 ☐ Casi siempre
3 ☐ Muchas veces
4 ☐ Algunas veces
5 ☐ Sólo alguna vez
6 ☐ Nunca

24. Durante las 4 últimas semanas, ¿cuánto tiempo estuvo muy nervioso?

1 ☐ Siempre
2 ☐ Casi siempre
3 ☐ Muchas veces
4 ☐ Algunas veces
5 ☐ Sólo alguna vez
6 ☐ Nunca

25. Durante las 4 últimas semanas, ¿cuánto tiempo se sintió tan bajo de moral que nada podía animarle?
1 ☐ Siempre
2 ☐ Casi siempre
3 ☐ Muchas veces
4 ☐ Algunas veces
5 ☐ Sólo alguna vez
6 ☐ Nunca

26. Durante las 4 últimas semanas, ¿cuánto tiempo se sintió calmado y tranquilo?
1 ☐ Siempre
2 ☐ Casi siempre
3 ☐ Muchas veces
4 ☐ Algunas veces
5 ☐ Sólo alguna vez
6 ☐ Nunca

27. Durante las 4 últimas semanas, ¿cuánto tiempo tuvo mucha energía?
1 ☐ Siempre
2 ☐ Casi siempre
3 ☐ Muchas veces
4 ☐ Algunas veces
5 ☐ Sólo alguna vez
6 ☐ Nunca

28. Durante las 4 últimas semanas, ¿cuánto tiempo se sintió desanimado y triste?
1 ☐ Siempre
2 ☐ Casi siempre
3 ☐ Muchas veces
4 ☐ Algunas veces
5 ☐ Sólo alguna vez
6 ☐ Nunca

29. Durante las 4 últimas semanas, ¿cuánto tiempo se sintió agotado?
1 ☐ Siempre
2 ☐ Casi siempre
3 ☐ Muchas veces
4 ☐ Algunas veces
5 ☐ Sólo alguna vez
6 ☐ Nunca

30. Durante las 4 últimas semanas, ¿cuánto tiempo se sintió feliz?
1 ☐ Siempre
2 ☐ Casi siempre

3 □ Muchas veces
4 □ Algunas veces
5 □ Sólo alguna vez
6 □ Nunca

31. Durante las 4 últimas semanas, ¿cuánto tiempo se sintió cansado?

1 □ Siempre
2 □ Casi siempre
3 □ Muchas veces
4 □ Algunas veces
5 □ Sólo alguna vez
6 □ Nunca

32. Durante las 4 últimas semanas, ¿con qué frecuencia la salud física o los problemas emocionales le han dificultado sus actividades sociales (como visitar a los amigos o familiares)?

1 □ Siempre
2 □ Casi siempre
3 □ Algunas veces
4 □ Sólo alguna vez
5 □ Nunca

POR FAVOR, DIGA SI LE PARECE CIERTA O FALSA **CADA UNA** DE LAS SIGUIENTES FRASES.

33. Creo que me pongo enfermo más fácilmente que otras personas.

1 □ Totalmente cierta
2 □ Bastante cierta
3 □ No lo sé
4 □ Bastante falsa
5 □ Totalmente falsa

34. Estoy tan sano como cualquiera.

1 □ Totalmente cierta
2 □ Bastante cierta
3 □ No lo sé
4 □ Bastante falsa
5 □ Totalmente falsa

35. Creo que mi salud va a empeorar.

1 □ Totalmente cierta
2 □ Bastante cierta
3 □ No lo sé
4 □ Bastante falsa
5 □ Totalmente falsa

36. Mi salud es excelente.

1 □ Totalmente cierta
2 □ Bastante cierta
3 □ No lo sé
4 □ Bastante falsa
5 □ Totalmente falsa

Fecha: ______________________________

Observaciones:

Cuestionary Reference:

Cuestionario de calidad de vida por síntomas urinarios (Alonso J, Prieto L, Antó JM. La versión española de SF 36 Health Survey (Cuestionario de Salud SF 36): in instrumento para la medida de resultados clínicos. Med Clin (Barc) 1995; 104: 771-76.): Adaptación del Prof Dr D José Antonio Mirón Canelo. Departamento de Medicina y Salud Pública de la Facultad de Medicina de Salamanca.
